# Multi-Robot Collaborative Mapping with Integrated Point-Line Features for Visual SLAM

**DOI:** 10.3390/s24175743

**Published:** 2024-09-04

**Authors:** Yu Xia, Xiao Wu, Tao Ma, Liucun Zhu, Jingdi Cheng, Junwu Zhu

**Affiliations:** 1 School of Information Engineering, Yangzhou University, Yangzhou 225127, China; 2 Advanced Science and Technology Research Institute, Beibu Gulf University, Qinzhou 535011, China

**Keywords:** visual SLAM, point and line features, map fusion, multi-robot mapping

## Abstract

Simultaneous Localization and Mapping (SLAM) enables mobile robots to autonomously perform localization and mapping tasks in unknown environments. Despite significant progress achieved by visual SLAM systems in ideal conditions, relying solely on a single robot and point features for mapping in large-scale indoor environments with weak-texture structures can affect mapping efficiency and accuracy. Therefore, this paper proposes a multi-robot collaborative mapping method based on point-line fusion to address this issue. This method is designed for indoor environments with weak-texture structures for localization and mapping. The feature-extraction algorithm, which combines point and line features, supplements the existing environment point feature-extraction method by introducing a line feature-extraction step. This integration ensures the accuracy of visual odometry estimation in scenes with pronounced weak-texture structure features. For relatively large indoor scenes, a scene-recognition-based map-fusion method is proposed in this paper to enhance mapping efficiency. This method relies on visual bag of words to determine overlapping areas in the scene, while also proposing a keyframe-extraction method based on photogrammetry to improve the algorithm’s robustness. By combining the Perspective-3-Point (P3P) algorithm and Bundle Adjustment (BA) algorithm, the relative pose-transformation relationships of multi-robots in overlapping scenes are resolved, and map fusion is performed based on these relative pose relationships. We evaluated our algorithm on public datasets and a mobile robot platform. The experimental results demonstrate that the proposed algorithm exhibits higher robustness and mapping accuracy. It shows significant effectiveness in handling mapping in scenarios with weak texture and structure, as well as in small-scale map fusion.

## 1. Introduction

Achieving safe autonomous mobility for robots in various environments requires solving the localization problem first, which involves determining the robot’s current position. Robots rely on sensors to perceive environmental information and utilize Simultaneous Localization and Mapping (SLAM) techniques to construct real-time maps while localizing themselves during movement. In well-known environments, mapping and localization are relatively straightforward, but in unknown or complex environments, accomplishing these tasks autonomously becomes significantly more challenging.

Current visual SLAM systems, particularly those relying on depth camera, monocular, or stereo setups, have shown great promise. Depth cameras directly compute obstacle distances using point cloud data, while monocular and stereo visual SLAM estimate object distances by tracking changes in image poses, enabling both localization and environmental modeling. These systems are advantageous over laser-based SLAM due to lower power consumption, simpler mechanical structures, and the ability to capture richer environmental details. However, despite significant advancements, these methods face critical challenges in large-scale indoor environments, especially in scenes with weak-texture structures. In such scenarios, the reliance on point features alone can lead to decreased mapping efficiency and localization accuracy. Moreover, the diversity and complexity of real-world environments often require scene-specific algorithmic adjustments, highlighting the limitations of existing visual SLAM techniques in handling varying textures and structures effectively.

To overcome these challenges, this paper proposes a multi-robot collaborative mapping algorithm based on point-line feature fusion. This approach integrates feature-extraction algorithms that combine point and line features with a map-fusion method grounded in scene recognition. The proposed method simultaneously extracts point-line features from image frames during visual odometry and uses scene recognition to determine overlaps in scene areas by assessing similarities between the current and historical frames. To maintain real-time performance, keyframes are selectively extracted rather than processing all image frames. The Perspective-3-Point (P3P) algorithm and Bundle Adjustment (BA) algorithm are then used to solve the relative pose-transformation relationships of multiple robots in overlapping scenes, facilitating effective map fusion.

Given that line features are more abundant in structured scenes, incorporating them alongside point features enriches the feature set, thereby improving mapping accuracy in weak-textured environments. Furthermore, the use of multiple robots allows for the decomposition of complex tasks into manageable subtasks [1], which can be handled collaboratively. This significantly enhances the overall system’s mapping efficiency, enabling it to tackle tasks that would be challenging for a single robot [2]. The proposed method was validated using publicly available datasets and tested on a mobile robot platform. Experimental results demonstrate that the enhanced algorithm can swiftly construct maps of weak-textured structured scenes, producing richer point cloud maps and thereby improving both mapping efficiency and localization accuracy. This approach addresses to some extent the limitations of existing visual SLAM methods in large-scale structured weak-texture environments.

## 2. Related Work

Visual SLAM techniques are categorized into direct methods [3,4,5] and feature-based methods [6,7,8]. Feature-based methods, such as PTAM algorithm [9] and the Oriented Fast and Rotated (ORB) algorithm [10], estimate camera pose by capturing environmental images, extracting and matching feature points, and minimizing reprojection errors. Despite improvements in ORB-SLAM [11] through integration with binocular and RGB-D cameras, reliance solely on point features remains insufficient in weak-texture environments, leading to increased feature errors and camera pose loss. In contrast, direct methods like Dense Tracking and Mapping (DTAM) [12] do not rely on environmental features; they enhance pose accuracy by computing depth maps but suffer from high computational complexity. Visual SLAM systems based on sparse direct methods [13] offer good real-time performance but are prone to losing tracked map points during large frame motions or illumination changes.

Recent advancements in SLAM have focused on addressing these limitations. Line features, as a higher-level geometric attribute, provide more robust tracking under varying illumination and viewpoints, overcoming some limitations of point feature extraction. For example, the line segment-parameterization [14] method simplifies SLAM computations. PL-SVO [15], an extension of SVO, incorporates line features to enhance performance in weak-textured environments but still struggles with dynamic illumination conditions. A. Pumarola [16] represents line segments using endpoints, converting them into feature points. They utilize the distance between endpoint-projected line segments to estimate camera pose errors, without considering loop closure detection. UPLP-SLAM [17] leverages multiple geometric feature types for effective navigation in structured environments. Xin Liu [18] enhances the frontend process of VINS fusion, proposing a real-time stereo visual-inertial SLAM system based on point-line features. Recent enhancements, such as Structure PLP-SLAM [19], combine point and line features for improved frontend pose tracking and backend map optimization.

Collaborative mapping among multi-robots [20,21] involves merging local submaps constructed by individual robots into a complete global map, where communication [22,23] plays a vital role in robot team cooperation. Compared to single-robot mapping, multi-robot collaborative mapping offers the advantage of faster environmental exploration. Methods like NeuralCoMapping [24] and MR-GMMapping [25] use multi-graph neural networks and Gaussian Mixture Models (GMMs) for more efficient map construction. Liwei Zhang [26] extends the RRT algorithm for optimization-based exploration, while MR-TopoMap [27] explores simultaneous construction of topological maps. LAMP 2.0 [28], building on LAMP [29], introduces an expandable multi-robot frontend and robust backend optimization for large-scale environments. While many successful algorithms exist for mapping using a single platform, collaborative mapping among robot teams remains a significant challenge.

Although previous SLAM systems such as PL-SVO and UPLP-SLAM have incorporated line features to address some issues, their performance in certain challenging conditions, such as dynamic illumination changes, remains suboptimal. And systems like LAMP 2.0 have introduced robust backends for map optimization in large-scale environments. Unlike these existing systems, our proposed approach enhances the frontend by simultaneously extracting both point and line features, which improves robustness in weak-textured environments, offering a more reliable solution to pose estimation. And our approach employing a map-fusion method based on scene recognition, which enhances the accuracy and reliability of collaborative mapping between robots, especially in indoor environments with weak textures.

## 3. Visual Mileage Calculation Method Based on Point and Line Features

**Figure 1:**
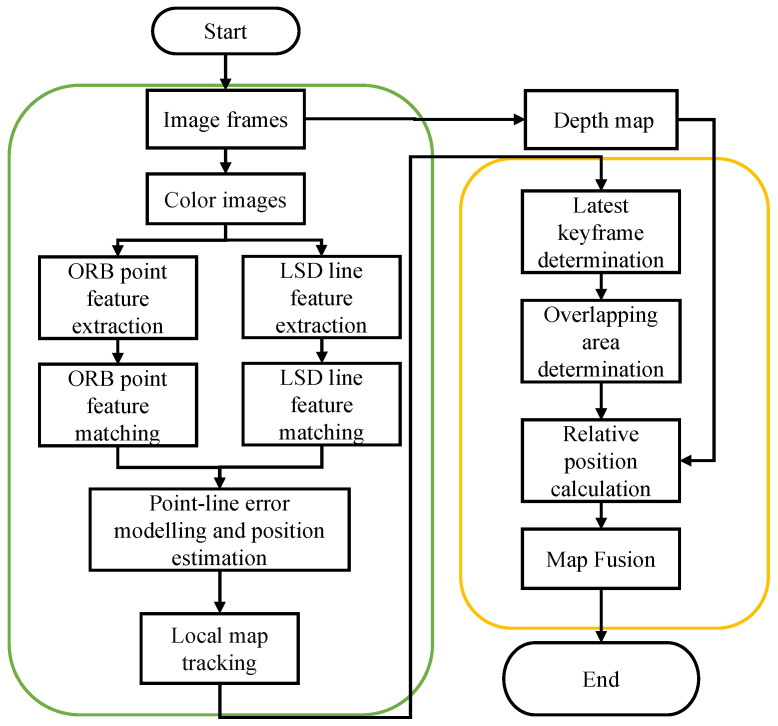
Visual odometry flowchart.

While feature-based methods are less likely to fall into local optima or lose track of map points under changes in illumination, their effectiveness may decrease in regions with insufficient texture. In contrast, line features exhibit more robust tracking performance under changes in illumination and viewpoint, thus compensating for the shortcomings of point features in low-textured areas. Therefore, this paper proposes a feature-extraction algorithm that integrates both point and line features. The improved complete visual odometry process is illustrated in Figure 1. After extracting and matching point-line features, separate error models for points and lines are constructed. The Jacobian matrix of the point-line reprojection model is then solved to compute the camera pose, ultimately completing the construction of the local map.

### 3.1. Feature Extraction and Matching of Point and Line Features

In this study, line features are introduced into the basic algorithm framework, integrating the Line Segment-Detection (LSD) algorithm and the Line Binary Descriptor (LBD) [30] to track and match line segment features, with outlier removal applied to line segments. The integration of line features significantly enhances localization accuracy and robustness, particularly in scenarios with sparse feature points but rich edge information. However, due to the discontinuity of depth maps generated by RGB-D camera at object boundaries, projecting features into the camera coordinate system based on depth values and camera intrinsics may lead to inaccurate projection due to depth errors. To address this issue, erroneous line features are removed based on the LSD line detection.

The algorithm for line detection is illustrated in Algorithm 1. Firstly, by setting a gradient threshold, pixels with gradients below this threshold are excluded from the construction range of the line support region. Subsequently, unused neighboring pixels in the region are recursively tested, and pixels with horizontal line angles equal to the region angle $\theta_{region}$ are added to the region. This process is repeated until no additional pixels are added to the region. If the image contains two straight edges and the angle formed is less than the tolerance τ, a situation arises where a rectangle encompasses both edges, severely affecting the accuracy of line segment extraction. Recognizing that the degree of approximation of a slightly curved edge by a series of straight edge segments is closely related to the density of alignment points, alignment point density is associated with the accuracy of curve approximation by line segments. If the image does not contain two straight edges, the rectangle can adapt well to the line support region, resulting in a high density of alignment points; however, if two straight edges exist in the image, the density of alignment points will be low. To address this issue, problematic line support regions are identified and divided into smaller regions. This is achieved by reducing the angle tolerance and region radius to segment the matrix. Firstly, the angle tolerance is reduced, adapting it to the region, and then the region-growing algorithm is employed to separate the two line segments. By extracting surrounding pixels within the original rectangle width and calculating the standard deviation of the horizontal line angles of these pixels, the new tolerance is set to twice the standard deviation. For greater accuracy, the method of reducing the region radius is adopted, progressively removing distant pixels until the alignment point density requirement is met.

Treating the pixel region as a rigid body, the “quality” of points can be measured by the magnitude of the pixel gradient. The centroid of the region is chosen as the center of the rectangle, and the first moment of inertia axis of the region serves as the principal direction of the rectangle. The width and length of the rectangle can be set to the minimum values that cover the region, and $c_{x}$ and $c_{y}$ can be calculated using the following equations:

(1) $$\begin{array}{c} \left\{\begin{array}{cc} c_{x} & =\frac{\sum_{j\in Region}G\left(j\right)\cdot x\left(j\right)}{\sum_{j\in Region}G\left(j\right)}\\ c_{y} & =\frac{\sum_{j\in Region}G\left(j\right)\cdot x\left(j\right)}{\sum_{j\in Region}G\left(j\right)} \end{array}\right. \end{array}$$

where G(j) represents the gradient magnitude of pixel *j*, while x(j) and y(j) denote the coordinates of the pixel. The angle of the principal matrix is the angle of the eigenvector associated with the minimum eigenvalue of the matrix. The detection results are determined by computing the number of false alarms for each rectangle, denoted as NFA:

(2) $$NFA\left(r\right)={\left(NM\right)}^{5/2}\gamma \times \left[\sum_{j=k}^{n}\left(\frac{n}{j}\right)p^{j}{\left(1-p\right)}^{n-j}\right]$$

where *N* and *M* denote the number of columns and rows of the scaled image, respectively.

**Algorithm 1:** Line segment-detection algorithm.

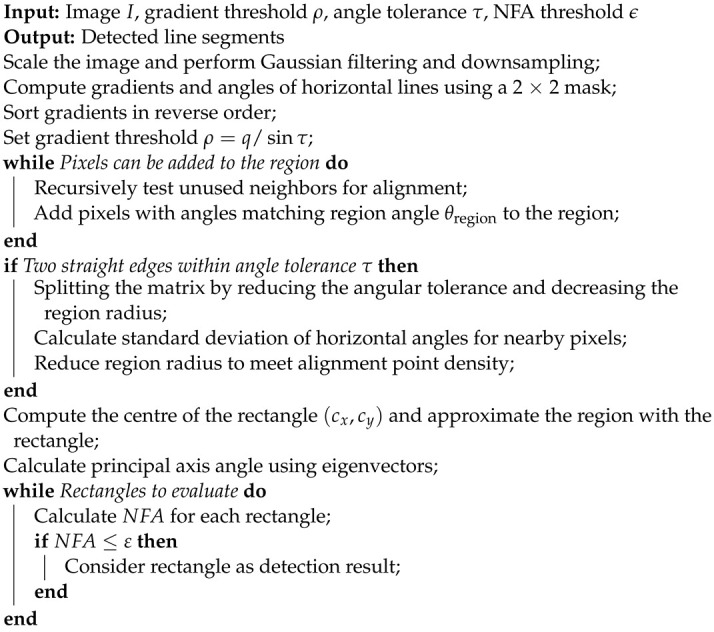



The extraction of line segments through the aforementioned steps suffers from inaccuracies in endpoint detection due to the lack of epipolar geometric constraints, resulting in segmentation errors and low discriminability of the same textured regions. To address these issues, we introduce local Gaussian weighting coefficients $f_{l}$ into the LBD descriptor, preserving the illumination and rotation invariance advantages of MSLD [31]:

(3) $$f_{l}\left(k\right)=\left(1/\sqrt{2\pi }\sigma_{l}\right)e^{-d_{k}^{2}/2\sigma_{l}^{2}},\sigma_{l}=w.$$

LBD creates a Line Support Region (LSR), where each column introduces a global Gaussian function $f_{g}$, mitigating the influence of distance on the descriptor:

(4) $$f_{g}\left(i\right)=\left(1/\sqrt{2\pi }\sigma_{g}\right)e^{-d_{i}^{2}/2\sigma_{g}^{2}},\sigma_{g}=0.5\left(m\cdot w-1\right).$$

To reduce boundary effects between each stripe, the local gaussian function $f_{l}$ is applied to adjacent stripes, and then the LSR is partitioned into a set of mutually parallel stripes $B_{1},B_{2},B_{3},\ldots ,B_{m}$. The LSR region is divided into *m* stripes, each with a pixel width of *w*. The stripe descriptor $BD_{j}$ is constructed using the pixel gradients of $B_{j}$ and its neighboring stripes $B_{j+1}$ and $B_{j-1}$. $BD_{j}$ is obtained from the mean vector $M_{j}$ and the standard deviation $S_{j}$ of the $BDM_{j}$ matrix:

(5) $$LBD={\left(BD_{1}^{T},BD_{2}^{T},\ldots ,BD_{m}^{T}\right)}^{T}.$$

In this paper, the Binary Robust Indepndent Elementary Features (BRIEF) descriptor is transformed into a 256-dimensional binary descriptor to enhance matching efficiency. Given the possibility of mismatches in line features, when the length of the two lines to be matched exceeds a certain fixed value or when the matching feature is within a distance *d* from the image boundary, they are excluded.

### 3.2. Error Model and Pose Estimation Based on Point-Line Features

After extracting point and line features from images, feature matching is performed between adjacent images, and the camera pose is finally estimated using the matching results. To address the reprojection error of 3D points and line segments onto the image plane, we employ the 3D-2D Perspective-n-Point (PnP) method [32] to estimate the camera motion. By considering coordinate transformations and the camera projection model, the reprojection error of point features is calculated, and nonlinear optimization of the objective function is conducted to determine the camera pose.

Assuming the homogeneous coordinates of a three-dimensional point *P* are $P={\left(X_{i},Y_{i},Z_{i},1\right)}^{T}$ and its 2D projection on the camera plane is denoted as $p_{i}={\left(u_{i},v_{i},1\right)}^{T}$, the Lie group representation of the camera pose R,t is denoted as *T*, and the camera intrinsics are represented by *K*. Thus, the relationship between pixel positions and spatial points can be expressed as:

(6) $$p^{\prime }=p_{i}*s_{i}=KTP.$$

After obtaining the error model for point features, we assume that the camera pose at time *i* is denoted as $T_{i}^{cw}$. At this time instant, the world coordinates of the *k*-th point are represented as $P_{k}^{w}$, with its reprojection error denoted as $e_{p}^{ki}$ and the corresponding pixel plane projection as $P_{c}^{ki}$. The reprojection error $E_{PL}$ for *m* points is expressed as:

(7) $$E_{PL}=\sum_{i=1}^{m}{∥e_{p}^{ki}∥}^{2}=\sum_{i=1}^{m}{∥P_{c}^{cw}-T_{i}^{cw}P_{k}^{w}∥}^{2}.$$

The camera pose is estimated using the Levenberg-Marquardt method, which involves computing the Jacobian matrix of the objective function with respect to the state variables. The Jacobian matrix of the reprojection error $e_{p}$ with respect to the incremental camera pose is expressed as:

(8) $$J_{p_{\xi }}=\frac{\partial e_{p}}{\partial \delta_{\xi }}=\frac{\partial e_{p}}{\partial p_{i}^{c}}\frac{\partial p_{i}^{c}}{\partial \delta_{\xi }}.$$

The Jacobian matrix of the reprojection error for three-dimensional spatial points is expressed as:

(9) $$J_{P_{p}}=\frac{\partial e_{P}}{\partial P_{i}^{w}}=\frac{\partial e_{P}}{\partial P_{i}^{c}}\frac{\partial P_{i}^{c}}{\partial P_{i}^{w}}.$$

The plucker matrix *E* is used to represent the geometric relationship in three-dimensional space. Assuming there are two points $S_{1}=\left(x_{1},y_{1},z_{1},1\right)$ and $S_{2}=\left(x_{2},y_{2},z_{2},1\right)$ in space, a straight line is determined with these two points as endpoints, with its unit direction vector denoted as *n*, and the coordinate origin as *O*. The vector normal to the plane passing through the coordinate origin is represented by *Z*. A straight line in space can be uniquely determined by *Z* and *n*. The unknowns in the plucker matrix *E* form a vector representation of the plucker coordinates *Z*. By projecting the plucker coordinates $Z_{w}$ of the line in the world coordinate system onto the camera coordinate system, the plucker coordinates $Z_{c}$ in the camera coordinate system are obtained:

(10) $$Z_{c}=\left(\begin{array}{cc} R_{cw} & t_{cw}\times R_{cw}\\ 0 & R_{cw} \end{array}\right)Z_{w}$$

where $R_{cw}$ denotes the rotation matrix, and $t_{cw}$ represents the translation vector. The pinhole model of the camera can compute the projection $l^{\prime }$ of the spatial line *L* on the pixel plane. The distance error $e_{l}$ between the actual matched line $l_{t}$ and the projected line segment $l^{\prime }$ can be expressed as:

(11) $$e_{l}=d\left(l,l^{\prime }\right)={\left(\frac{S_{1}^{T}l^{\prime }}{\sqrt{\left(l^{2}+{l^{\prime }}^{2}\right)}},\frac{S_{2}^{T}l^{\prime }}{\sqrt{\left(l^{2}+{l^{\prime }}^{2}\right)}}\right)}^{T}.$$

The Jacobian matrix of the line’s reprojection error $e_{l}$ with respect to the camera motion increment can be expressed as:

(12) $$J_{l_{\xi }}=\frac{\partial e_{l}}{\partial \delta_{\xi }}=\frac{\partial e_{l}}{\partial L^{\prime }}\frac{\partial L^{\prime }}{\partial E_{c}}\frac{\partial E_{c}}{\partial \delta_{\xi }}$$

where $E_{c}$ represents the plucker matrix of the line in the camera coordinate system, and $L^{\prime }=K^{\prime }n_{c}$ denotes the projection of the spatial line onto the camera plane. The Jacobian matrix of the line segment’s reprojection error with respect to the orthogonal increment can be expressed as:

A spatial line can be represented in orthogonal form using four parameters:

(13) $$U=\left[\begin{array}{ccc} u_{11} & u_{12} & u_{13}\\ u_{21} & u_{22} & u_{23}\\ u_{31} & u_{32} & u_{33} \end{array}\right],W=\left[\begin{array}{cc} w_{1} & -w_{2}\\ w_{3} & w_{4} \end{array}\right].$$

According to the equation of a spatial line, we obtain:

(14) $$\left\{\begin{array}{cc} L_{c} & =T_{c}^{w}L_{w}\\ \frac{\partial L_{c}}{\partial L_{w}} & =\frac{\partial T_{c}^{w}}{\partial L_{w}}=T_{c}^{w}\\ \frac{\partial L_{w}}{\partial \delta_{\xi }} & =\left[\begin{array}{cc} -{\left(w_{1}u_{1}\right)}^{\wedge } & -w_{2}u_{1}\\ -{\left(w_{2}u_{2}\right)}^{\wedge } & -w_{1}u_{2} \end{array}\right] \end{array}\right..$$

The translation matrix and rotation matrix of the camera can be obtained using the derived Jacobian matrix, resolving the nonlinear optimization problem, thus computing the camera pose.

### 3.3. Building Local Maps

The estimation of camera pose relies on the previous moment. If pose optimization is performed at every moment, the accumulated errors over prolonged motion would increase computational complexity. Thus, it is necessary to select representative keyframes for optimization. This paper adopts a keyframe selection method based on photogrammetry principles. After selecting the current frame as a keyframe, the local map construction is completed through three steps: generation, deletion, and building of a co-visibility graph for spatial points and lines. Spatial points and lines generated in the new keyframe are added as landmarks to the local map, and the depth information obtained from the depth camera is used to recover spatial points and lines. For depth values outside the range of the depth camera, feature matching with neighboring keyframes is required, followed by depth value calculation through triangulation.

If newly generated spatial points and lines are only observed by the current keyframe and not by other keyframes, it would lead to significant computational and storage waste. Therefore, spatial points and lines need to be filtered. Keyframes meeting the criteria must meet the following requirements: (1) Spatial points and lines generated by new keyframes should be observed by at least three keyframes; (2) The projection of new spatial points and lines should be within the field of view of keyframes, and at least 30% of the keyframes should be able to track the spatial points and lines through feature matching. Landmarks that meet the criteria are added to the local map, while those that do not are deleted.

After all landmarks of the current frame are added, firstly, all landmarks are read, and the observation information and landmarks of co-visible old landmarks are updated. Landmarks without common observation information are stored. Then, all observation information of the current frame’s landmarks is extracted. Each landmark contains multiple observation information, with each observation corresponding to a keyframe. The number of observations between the current keyframe and all keyframes is recorded and sorted. Keyframes with a common observation count exceeding 20 are selected and stored in the co-visibility graph of the current keyframe. If no keyframe meets the criteria, keyframes capable of detecting more co-visible observation information are added to the co-visibility graph, followed by the establishment of a local map through the co-visibility graph. Next, the pose of keyframes and landmarks is optimized using BA. The keyframes’ poses and the positions of spatial points $p_{i}$ and spatial lines $L_{i}$ in the local map are optimized, reducing the scale of the problem and considering more pose constraints to improve system accuracy. The objective function is as follows:

(15) $$\left\{T_{k}^{*},p_{i}^{*},L_{j}^{*}\right\}=argmin\left(\sum_{k,i}∥ep_{k,i}{∥}^{2}+\sum_{k,j}{∥el_{k,j}∥}^{2}\right).$$

## 4. Map-Fusion Algorithm Based on Scene Recognition

This paper utilizes visual information and visual bag of words to associate data between two robots. Robot 1, as the fusion end, is responsible for storing the visual bag of words between the two robots, while robot 2 acts as the client. The image frames, related pose information, and local map data obtained by robot 2 are transmitted via topics using the TCP/IP protocol, and the fusion end can subscribe to this information at any time. During the collaborative map construction process, each robot conducts its own loop closure exploration. In the collaborative process, different robots traverse the same scenes, leading to scene overlap. Based on this, this paper proposes a map-fusion method based on scene recognition, where the local submaps for map fusion are composed of image frame sequences collected by robots, and the scene-recognition process involves detecting the similarity between each pair of image frames.

### 4.1. Keyframe Selection

The process of scene recognition can be viewed as detecting the similarity between image frames of two robots. However, using all image frames for determination would result in a huge computational load, affecting system real-time performance, and even leading to system crashes. Therefore, it is necessary to extract keyframe information from the images. The candidate keyframe selection method in this paper includes: (1) Insertion of frames beyond 20 frames from the last keyframe or when the local map construction thread is in idle state; (2) The current frame tracks at least 50 feature points and 15 spatial lines; (3) The proportion of features in the current frame compared to those in the reference keyframe does not exceed 75%. We adopt a keyframe selection method based on geometric and photogrammetric principles, namely the Image Network Designer (IND) method, for further filtering of selected candidate frames. This method fully utilizes adaptive thresholds to determine whether to select keyframes.

**Figure 2:**
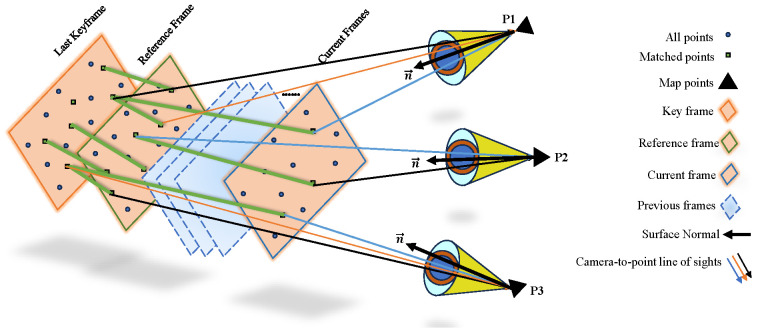
Schematic diagram of keyframe selection method.

For distant keyframes, we can observe the number of intersection points in each frame. For each 3D point, we define a four-quadrant spatial cone with an angle of 10 degrees, as shown in Figure 2. The choice of a 10-degree angle is determined based on the maximum inclination angle at which most keypoint-detection algorithms can find corresponding points in the image. For each frame, we compute the points where the “camera-to-point line-of-sight region” has changed since the last keyframe and use an adaptive threshold to determine if the change in quantity is significant. The adaptive threshold is defined based on the total number of points in each frame, the number of matched points, and the number of points in the cone region that have already changed. As frames move from the last keyframe to the current frame, the angle changes, altering the direction of the line-of-sight vector region. The adaptive threshold models frame displacement based on the number of line-of-sight-changed points to facilitate keyframe selection decisions:

(16) $$Th_{adaptive}=\left(1+\varepsilon \theta -\beta -\alpha \right)\times Th_{initial}$$

where

(17) $$Th_{initial}=0.5\times \left(\frac{M_{c}D_{r}}{M_{r}}+\frac{A_{c}D_{r}}{A_{r}}\right)$$

and $Th_{initial}$ denotes the initial value of the adaptive threshold, which is the average of initial values obtained from all points and matched points to accommodate the influence of two parameters. $M_{r}$ represents the number of points in the current frame that have correspondences in the last keyframe, $M_{c}$ is the number of points in the current frame corresponding to the previous keyframe, $D_{r}$ is the number of points in the reference frame whose cone regions have changed from the last keyframe, and $A_{c}$ and $A_{r}$ represent all points in the current frame and the reference frame, respectively. This threshold assumes that the state of the current frame is similar to the reference frame and simulates the initial threshold for change count using the number of corresponding points and points whose line of sight has changed. Finally, this initial threshold will be closer to the actual value, with the coefficient depending on the geometric conditions of the frame. If the current frame is similar to the reference frame, how many “cone regions of the current frame” should have changed from the last keyframe to the current frame.

When more than half of the matching points in the cone region undergo changes, triangular geometry is used to select keyframes, and the adaptive threshold is sufficient to generate acceptable values under any frame conditions. The adaptive threshold criterion models only the number of changes compared to the previous keyframe. As these changes increase, the probability of a positive response to the keyframe selection criteria increases, so it is necessary to control the number of changes to avoid misalignment between the current frame and the previous keyframe. Considering the stability of the framework, the Equilibrium Of Center Of Gravity (ECOG) criterion must also be used to determine whether points are evenly distributed throughout the image. Therefore, this paper designs a criterion to control the stability of the framework by considering the distribution of effective points. Additionally, keyframes with evenly distributed effective points must be selected to create dense and accurate point clouds. This criterion must be established simultaneously with the previous criteria. If the aforementioned conditions are met, the following calculations are performed to improve the algorithm’s performance: the image is divided into a 33 grid, and points in each grid where the cone region changes by more than δ degrees are considered effective points. For each image, this process generates a 33 matrix, and the ideal state is when the two largest values in the matrix are farthest apart. The ECOG criterion outputs a value of 1 in this ideal scenario, and between 0 and 1 under other conditions:

(18) $$ECOG=\frac{d\times {max}_{2}}{2\sqrt{2}{max}_{1}}$$

where

(19) $$d=\sqrt{{\left(r_{{max}_{2}}-r_{{max}_{1}}\right)}^{2}+{\left(c_{{max}_{2}}-c_{{max}_{1}}\right)}^{2}}$$

and ${max}_{1}$ represents the maximum value in the matrix, ${max}_{2}$ denotes the second largest value following ${max}_{1}$, *d* stands for distance, *r* and *c* respectively denote the row and column components. The smaller the distance *d* between two matrix elements, the smaller the value of ECOG.

As the distance decreases, the accumulated amount of points on one side of the matrix increases, and geometrically, the center of mass of the matrix will not be located at the center. The threshold th of ECOG can be controlled by computing the center of mass of the matrix:

(20) $$th=\sqrt{X_{c}^{2}+Y_{c}^{2}}$$

where $X_{c}$ and $Y_{c}$ represent the components of the matrix center of mass. As long as this criterion is below the threshold, the distribution of points in the frame is appropriate, and the frame has sufficient stability to be selected as a keyframe. If it also satisfies the adaptive threshold for points in the changing region, the frame will be selected as a keyframe.

### 4.2. Visual Bag of Words Method for Determining Areas of Overlap

Visual Bag of Words (BoW) model [33] is adopted in this paper for overlap area determination, which simplifies computational complexity during prolonged and extensive robot motion. ORB point features and LSD line features are extracted, and BRIEF and LSD descriptors are computed. Additional descriptor bits are added to prevent the mixing of point and line features, with point and line features labeled as 0 and 1, respectively. Initially, k-means clustering is performed on the first layer of descriptors to calculate weights and generate the second layer. Subsequent clustering is applied to the second layer until no further clustering is possible, resulting in the formation of a visual dictionary. Subsequently, the real-time image scenes captured during robot motion are matched with visual words in the dictionary to determine scene overlap. The robot continuously acquires new keyframes at the current time during mapping. After obtaining a new keyframe, visual features in the visual dictionary are trained, and k-means clustering is performed to generate binary visual vectors. The generated binary visual vectors are numbered and classified and stored in the dictionary, which consists of a tree structure with *d* layers, each containing *k* nodes, with “word” as the leaf node. The continuous process of environment mapping by the robot also implies the continuous update of the dictionary tree.

In this paper, reverse indexing is used to determine the matching between new keyframes and existing keyframes in the dictionary. Firstly, the words contained in the image are determined by extracting features from the new keyframe. Then, based on the obtained words, the corresponding positions in the index are found, which contain a set of images with that word. Finally, when an image with a certain number appears once, the cardinality of the corresponding voting array is incremented by one, and the keyframe with the most votes is selected as the candidate keyframe. The selected candidate keyframes do not need to calculate the similarity with all keyframes, which accelerates the matching process. TF-IDF (Term Frequency-Inverse Document Frequency) is used in this paper to compute the weights of words in the visual bag of words.

Assuming there are *N* features in the visual dictionary, $n_{w}$ features in a visual word, $n_{d}$ is the total number of visual words in the image, and $n_{wd}$ represents the number of occurrences of the visual word *w* in the current image. The weight $\beta_{w}$ of the visual word *w* can be represented as:

(21) $$\beta_{w}=TF\times IDF=\frac{n_{wd}}{n_{d}}log\frac{N}{n_{w}}$$

where $log(N/n_{w})$ represents the inverse document frequency, indicating that the more frequent the occurrence of visual word *w* in the image, the lower its weight. Meanwhile, $n_{wd}/n_{d}$ represents the document frequency, indicating that the more frequent the occurrence of visual word *w* in the image, the higher its weight. When the visual word *w* appears in image *I*, its weight is 0. After introducing weights, the vector of image *I* can be represented as:

(22) $$\begin{array}{c} v=\left\{\left(w_{p1},\beta_{w_{p1}}\right),\ldots ,\left(w_{Np},\beta_{w_{Np}}\right),\ldots ,\right.\\ \left.\left(w_{l1},\beta_{w_{l1}}\right),\ldots ,\left(w_{Nl},\beta_{w_{Nl}}\right)\right\} \end{array}$$

where $w_{p}$ represents the visual word for point features, while $w_{l}$ represents the visual word for line features.

Once images are represented as vectors, we measure the similarity between images using the Hamming distance between vectors. Assuming two word vectors are $\nu_{m}$ and $\nu_{n}$, the Hamming distance can be expressed as:

(23) $$s\left(v_{m},v_{n}\right)=1-\frac{1}{2}\left|\frac{v_{m}}{\left|v_{m}\right|}-\frac{v_{n}}{\left|v_{n}\right|}\right|.$$

**Figure 3:**
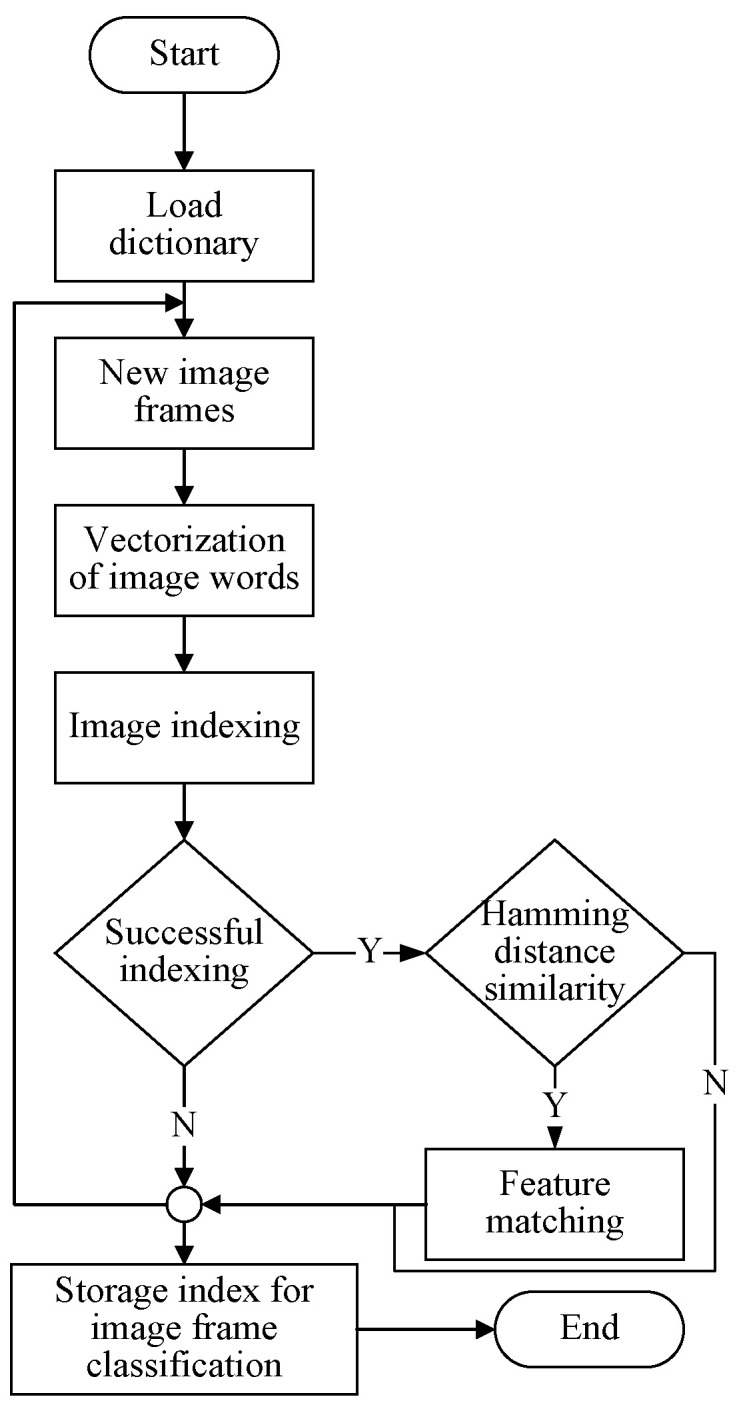
Image-similarity-recognition flowchart.

The greater the Hamming distance $s(\nu_{m},\nu_{n})$, the higher the similarity between images. In this paper, a threshold η is set, and if $s(\nu_{m},\nu_{n})>\eta$, the frame is considered a similar frame. When the number of similar frames reaches a certain threshold, it can be inferred that there is an overlapping area between the two moving robots, and the relevant relationship between the two poses can be established, followed by relative pose estimation. Subsequently, local submaps are transmitted and map fusion is performed. Figure 3 illustrates the recognition process.

### 4.3. Relative Pose Computation

This section aims to address the problem of relative pose transformation between two robots to establish the pose relationship between them. When robot 1 reaches a certain similar scene, while robot 2 may not be in that scene, it is necessary to transform the pose of robot 2 to that scene during this period. Assuming robot 1 arrives at scene A at time *t*, and robot 2 also arrives at scene A at time *k*, but arrives at scene B at time *t*. Now, suppose the relative pose of robot 2 at time *t* is $\left(r_{t}^{2},q_{t}^{2}\right)$, then the pose transformation of robot 2 to scene A can be represented as:

(24) $$\left\{\left[\begin{array}{cc} R_{k+1}^{2} & t_{k+1}^{2}\\ 0 & 1 \end{array}\right],\left[\begin{array}{cc} R_{k+2}^{2} & t_{k+2}^{2}\\ 0 & 1 \end{array}\right],\ldots \left[\begin{array}{cc} R_{t}^{2} & t_{t}^{2}\\ 0 & 1 \end{array}\right]\right\}$$

(25) $$T_{i}^{2}=\left[\begin{array}{cc} R_{t}^{2} & t_{t}^{2}\\ 0 & 1 \end{array}\right],i=k+1,k+2,\ldots ,t.$$

The transition matrix of robot 2 between keyframes at time *t* and *k* can be expressed as:

(26) $$T_{k\to t}^{2}=T_{k+1}^{2}T_{k+2}^{2}\ldots T_{t}^{2}$$

Through this, we can obtain the pose-transformation matrix of robot 2 relative to robot 1 in the local coordinate system at time *k*:

(27) $$\left[\begin{array}{cc} C_{k}^{2} & r_{k}^{2}\\ 0 & 1 \end{array}\right]={T_{k\to t}^{2}}^{-1}\left[\begin{array}{cc} C_{t}^{2} & r2_{t}\\ 0 & 1 \end{array}\right]$$

where *C* represents the pose of robot 2. Through the aforementioned process, the pose information correlation between the two robots in the shared scene A can be determined. The P3P method is employed in this paper to compute the initial solution for obtaining the relative pose relationship between robot 1 and robot 2 at time *k*. The constructed feature point map is visualizable, and its three-dimensional information and camera intrinsic matrix are known. However, when there are a large number of matched point pairs, P3P may fall into local optima, leading to incorrect inter-frame motion estimation. Therefore, it is necessary to combine BA optimization to improve the accuracy of the solution.

**Figure 4:**
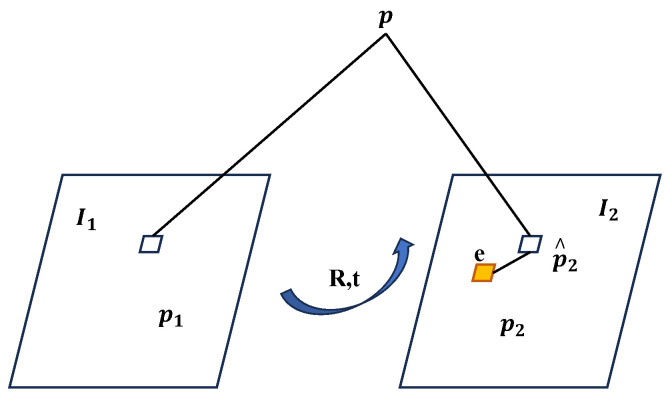
Reprojection errors.

Spatial coordinates of map points may exhibit a distance discrepancy between the reprojection on images under relative motion and the actual pixel projection points. The objective of BA optimization is to minimize this distance discrepancy, thereby reducing reprojection errors, as illustrated in Figure 4.

Where *p* represents a map point, $I_{1}$ and $I_{2}$ denote the preceding and subsequent frames, $p_{1}$ and $p_{2}$ are the actual projected pixels of map point *p* on the two frames, ${\hat{p}}_{2}$ is its reprojected point, *e* is the error value of pixel coordinates, and *R* and *t* are the inter-frame motion parameters. The ideal scenario can be described as follows:

(28) $$\left\{\begin{array}{cc} S_{1}\left[\begin{array}{c} X_{1}\\ 1 \end{array}\right] & =M_{in}X\\ S_{2}\left[\begin{array}{c} X_{2}\\ 1 \end{array}\right] & =M_{in}\left(RX+t\right) \end{array}\right.$$

where $X_{1}$ and $X_{2}$ represent the pixel coordinates of image points $p_{1}$ and $p_{2}$, $S_{1}$ and $S_{2}$ are the depth values of point *p* in the two frames, and $M_{in}$ is the camera’s intrinsic matrix. Successful feature point matches satisfy Equation (28), and optimization yields the following equation:

(29) $$\min J=\min_{R,t,X}\sum_{j=1}^{N}{∥S_{2}^{j}X_{2}^{j}-M_{in}\left(RX_{j}+t\right)∥}^{2}$$

where *N* is the number of successfully matched point pairs. Solving the above equation yields the optimal relative motion solution and the optimized coordinates of map points.

### 4.4. Map Fusion

Once robots detect scene overlap, the pose relationship between the two robots can be determined, data information can be integrated, and map fusion can be completed. Robot 1 and robot 2 each construct their own local maps and establish visual dictionary libraries containing their own poses and sensor information. When a robot obtains a new keyframe, its features are compared and verified one by one with the content in the visual dictionary library. After successfully identifying similar scenes, pose estimation is performed to obtain the relative pose relationship between the two robots. Map fusion can be performed at any time and between any robots, utilizing the relative pose relationship between robots to achieve coordinate synchronization. If robot 1 is considered the upper-level unit, map fusion is performed in robot 1’s local coordinate system. This process merges robot 2’s local map with the map constructed by robot 1 through the relative pose relationship R,t, as expressed below:

(30) $$\left[\begin{array}{c} P_{j}^{2^{\prime }}\\ 1 \end{array}\right]=\left[\begin{array}{cc} R & t\\ 0 & 1 \end{array}\right]\left[\begin{array}{c} p_{j}^{2}\\ 1 \end{array}\right],j=1,2,\ldots ,m^{2}$$

where $p_{j}^{2}$ and $p_{j}^{2^{\prime }}$ represent the poses of robot 2 at the two moments, and $m^{2}$ is the number of map features in the map constructed by robot 2.

The fused map is in the coordinate system at time *k* and needs to undergo the following transformations to convert it to robot 1’s local map coordinate system to obtain the complete map:

(31) $$\left[\begin{array}{c} p_{j}^{1}\\ 1 \end{array}\right]=P_{0}^{1}P_{2}^{1}\ldots P_{k}^{1}\left[\begin{array}{c} p_{j}^{1}\\ 1 \end{array}\right],j=1,2,\ldots ,m^{1}+m^{2}$$

where $p_{0}^{1}\ldots p_{k}^{1}$ is the matrix expression of local coordinates accumulated by robot 1 through relative coordinates, and $m^{1}+m^{2}$ represents the number of features in the map after fusion.

The map-fusion framework is depicted in Algorithm 2, which constructs global maps from the respective local maps of the robots. Robot 1 generates global poses, current image frames, and visual dictionary library 1, while robot 2 generates local poses, current image frames, and visual dictionary library 2. Determining whether there is scene overlap in the visual dictionary library essentially involves assessing the similarity between the current frame and historical frames. Overlap area determination is performed using the BoW model; if overlap exists, relative poses are calculated and maps are fused; if no overlap exists, the next frame is evaluated. Solving the relative pose between two robots is necessary as the relative pose transformation exists between the two robots.

**Algorithm 2:** Map-fusion algorithm based on point and line features.

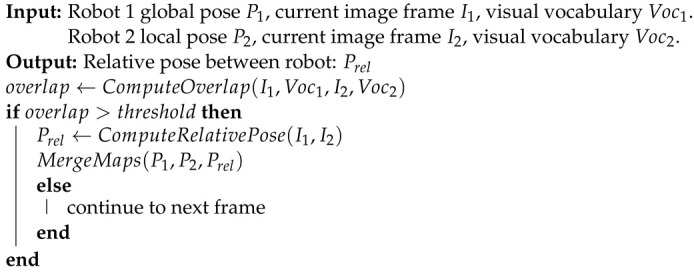



## 5. Experiments

### 5.1. Details

**Figure 5:**
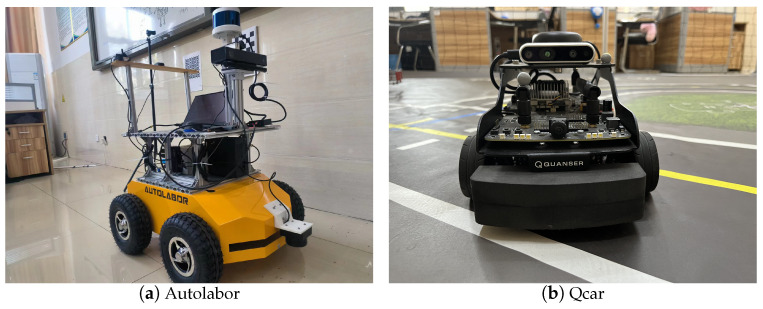
Mobile robotics platform.

Experimental validation with heterogeneous mobile robot platform the experiments in this paper are conducted using a heterogeneous mobile robot platform composed of Autolabor Pro1 and Qcar. The PC system of the mobile robot platform runs Ubuntu 18.04 ROS Melodic, and the reading and publishing of sensor topic information, as well as the execution of visual SLAM algorithms, are all performed in the ROS system. The hardware platform of the mobile robot consists mainly of sensor modules, peripheral modules, upper computer modules, and mobile chassis modules, with communication between heterogeneous robots achieved via WiFi. The devices of the mobile robot platform used in this paper are depicted in Figure 5.

Experimental dataset selection the TUM dataset is selected as the test dataset. The TUM dataset is a well-known RGB-D dataset collected by moving robots, with variations in motion speed and frame count, facilitating error analysis. We primarily select the Structure_texture, Structure_notexture, and Nostructure_notexture datasets from it. Experimental setup the experiments are conducted in the same environment, and the final trajectories are aligned with the ground truth trajectories using SE3 [34]. Experimental comparative analysis is performed between the multi-robot collaborative mapping algorithm based on point-line fusion proposed in this paper and the ORB-SLAM3 algorithm and ORB-SLAM2 algorithm.

Performance evaluation the results of testing the dataset are compared with the provided ground truth measurement data. The motion trajectories of the camera in each coordinate system, i.e., the estimated values and the ground truth values in the dataset, are compared. The evaluation is conducted using the Root Mean Square Error (RMSE) formula:

(32) $$X_{\mathrm{RMSE}}=\sqrt{\left(\frac{\sum_{i=1}^{n}{\left(X_{obs,i}-X_{\left(mod,i\right)}\right)}^{2}}{n}\right)}$$

where $X_{obs,i}$ represents the true coordinates of the camera position at time *i*, and $X_{mod,i}$ represents the estimated coordinates of the camera position at time *i*. The Nostructure_texture dataset experienced tracking failure during tracking. The RMSE is calculated to evaluate the performance of the three algorithms on the remaining datasets.

### 5.2. Performance

#### 5.2.1. Experimental Testing of Enhanced Point-Line Feature-Extraction Algorithm

**Figure 6:**
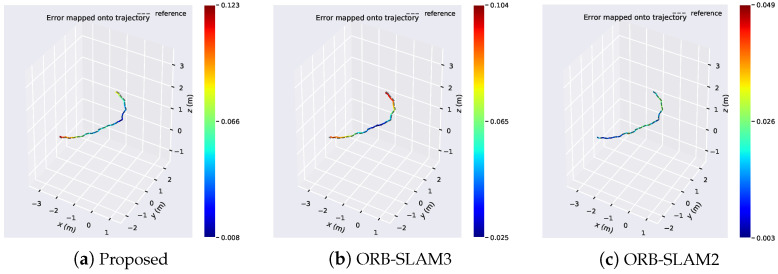
Comparison of error distribution of algorithms in Structure_texture dataset.

Figure 6 compares the error distributions of the proposed algorithm with ORB-SLAM3 and ORB-SLAM2 algorithms on the Structure_texture dataset. It can be observed that the error distribution of the proposed algorithm ranges from 0.008 to 0.123, while for ORB-SLAM3, it ranges from 0.025 to 0.104, and for ORB-SLAM2, it ranges from 0.003 to 0.049. Although the error distribution of the proposed algorithm is not as competitive as the comparative algorithms, its minimum error outperforms ORB-SLAM3. In scenes with structured and textured features, the proposed algorithm excels in extracting edge structural features, which the comparative algorithms fail to do due to the lack of line feature extraction.

**Figure 7:**
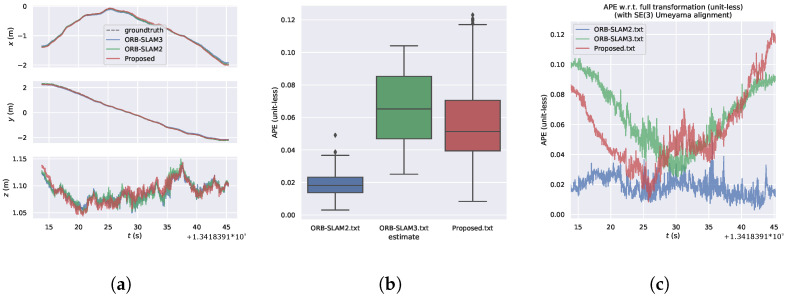
Visual analysis of the algorithm’s APE results in the Structure_texture dataset. (**a**) Algorithmic trajectories and changes in the position of the axes. (**b**) Boxplot of APE. (**c**) Changes in APE over time.

Figure 7 presents the visual analysis of Absolute Pose Error (APE) results of the algorithm on the Structure_texture dataset. Figure 7a provides a detailed comparison of the algorithm’s positional variations over time in the x, y, and z axes. Comparative analysis reveals close alignment with the ground truth trajectory for all three algorithms, with similar performance observed in scenes with structured and textured features. Figure 7b depicts the box plot of APE, while Figure 7c illustrates the variation of APE over time. It can be observed that in scenes with structured and textured features, although the relative error of the proposed algorithm is slightly inferior to the comparative algorithms, its overall performance is marginally better than that of the ORB-SLAM3 algorithm.

Table 1 presents the accuracy results of the algorithm on the Structure_texture dataset. The RMSE of our proposed algorithm is only 0.008 m different from ORB-SLAM3 and 0.041 m different from ORB-SLAM2, demonstrating slightly superior performance compared to ORB-SLAM3.

**Figure 8:**
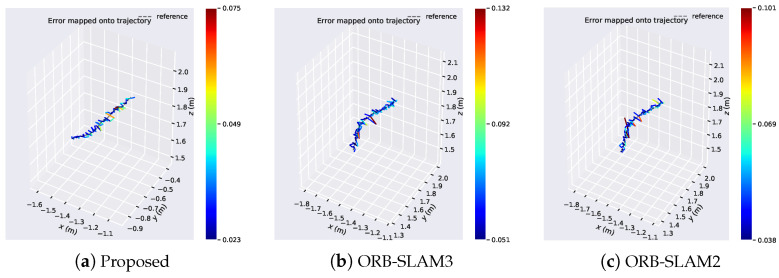
Comparison of error distributions of algorithms in the Nostructure_notexture dataset.

Figure 8 illustrates the error distribution comparison of the algorithm on the Nostructure_notexture dataset. It can be observed that in environments without structure and texture, the error distribution of our proposed algorithm is more concentrated compared to ORB-SLAM3 and ORB-SLAM2, with smaller maximum error values. All three algorithms exhibit a reduction in extracted features compared to other datasets, with our proposed algorithm extracting richer features compared to ORB-SLAM3.

**Figure 9:**
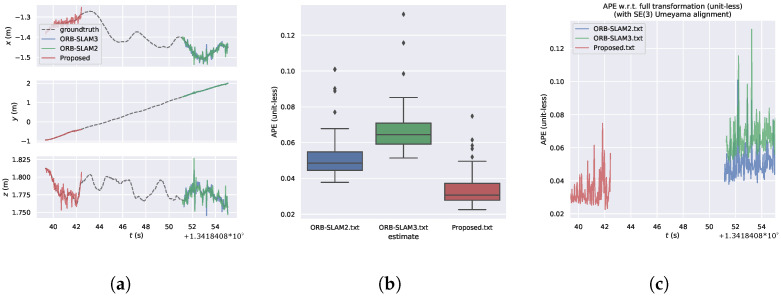
Algorithm for visual analysis of APE results in the Nostructure_Notexture dataset. (**a**) Algorithmic trajectories and changes in the position of the axes. (**b**) Boxplot of APE. (**c**) Changes in APE over time.

Figure 9 presents the visual analysis of the APE results of the algorithm on the Nostructure_notexture dataset. Figure 9a provides a detailed comparison of the x,y,z positions of the algorithm over time, indicating that our proposed algorithm can achieve feature matching approximately 10 s earlier. Figure 9b shows the box plot of APE, while Figure 9c demonstrates the change of APE over time, revealing that our proposed algorithm exhibits the smallest relative error in APE compared to the other two algorithms.

Table 2 provides the accuracy comparison results of the algorithm. Comparing the RMSE values, it can be observed that our proposed algorithm exhibits the best performance in terms of RMSE, indicating its advantage over ORB-SLAM3 and ORB-SLAM2 in environments with weak structure and texture.

**Figure 10:**
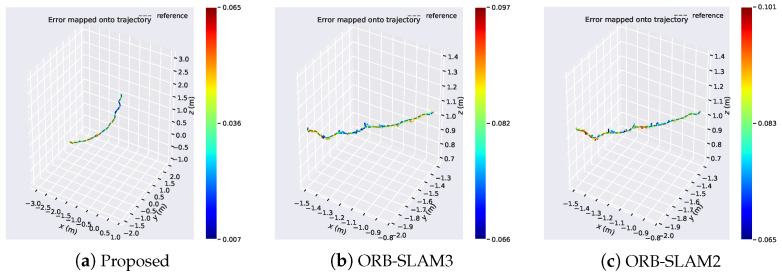
Comparison of error distribution of algorithms in Structure_notexture dataset.

Figure 10 presents the error distribution of the algorithm in the Structure_notexture dataset. The last dataset chosen is the Structure_notexture dataset, which corresponds to scenes with structured weak texture, typical of most indoor artificial environments. The algorithm proposed in this paper is primarily designed for such environments. A comparison reveals that in this setting, our algorithm exhibits errors ranging from 0.007 to 0.065, while the error distribution of ORB-SLAM3 ranges from 0.066 to 0.097, and ORB-SLAM2 ranges from 0.065 to 0.101.

**Figure 11:**
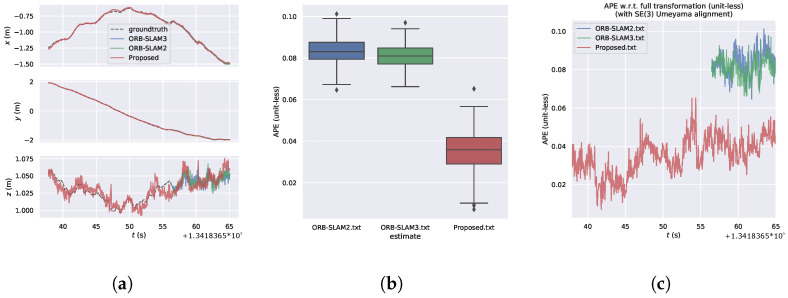
Algorithm for visual analysis of APE results in the Nostructure_Notexture dataset. (**a**) Algorithmic trajectories and changes in the position of the axes. (**b**) Boxplot of APE. (**c**) Changes in APE over time.

From Figure 11, it can be observed that our algorithm exhibits the smallest relative error in APE (unit-less) compared to other algorithms. Figure 11a provides a comparative analysis of the x,y,z positions of the algorithm over time. Our algorithm achieves initialization around 35 s, while ORB-SLAM2 and ORB-SLAM3 algorithms achieve initialization around 56 s. Our algorithm closely approximates the ground truth in terms of x,y,z positions. Even under conditions of structured weak texture, our algorithm nearly perfectly matches the true trajectory, outperforming other algorithms. Figure 11b shows the box plot of APE, while Figure 11c illustrates the change of APE over time, where our algorithm exhibits the best performance compared to the other two algorithms.

Table 3 provides the accuracy results of the algorithm on the Structure_notexture dataset. Comparing the RMSE values, it can be observed that our proposed algorithm exhibits the best performance in terms of RMSE, indicating its advantage over other algorithms in the Structure_notexture environment.

As this paper primarily focuses on research conducted in structured environments with weak texture, the algorithm’s RMSE was compared in such scenarios to better verify its localization accuracy. The comparative results from Table 4 indicate that under the same dataset conditions, the RMSE of our algorithm is smaller compared to ORB-SLAM2 and ORB-SLAM3 algorithms. Hence, it can be inferred that our algorithm demonstrates good robustness in scenes with pronounced structures and weak textures. The proposed algorithm, which integrates point and line features, significantly enhances localization accuracy. In indoor environments with structured weak textures, our point-line-fusion-based approach achieves more precise localization compared to methods relying solely on point features.

#### 5.2.2. Map-Fusion Experiment

**Figure 12:**
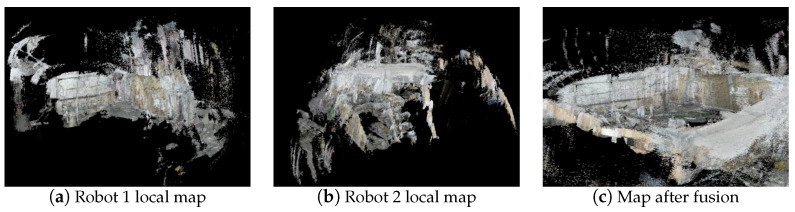
Robot map fusion.

In our experimental setup conducted in a real environment, two robots were deployed simultaneously to cooperatively explore uncharted territories. Robot 1 and robot 2 were dispatched concurrently, with robot 1 traveling from Scene A to Scene B while robot 2 departed from Scene B, each independently constructing local maps. During robot 1’s traversal, utilizing a bag of words model, keyframes were examined for scene detection. Upon detecting a similar frame to the current keyframe (in this case, robot 1 identified a similar frame at location B), indicating scene overlap with robot 2, the relative pose relationship between the two robots was solved, facilitating map fusion. Figure 12 illustrates the local maps constructed by the robots and the fused map. It is evident that the local maps constructed by both robots are quite comprehensive, with features such as whiteboards and signs being well-represented. Map fusion occurs within the overlapping region of the two robots. When one robot detects this overlap, map fusion between the two robots ensues, resulting in a global map that effectively integrates the locally constructed maps.

To validate the significant enhancement in mapping efficiency afforded by multi-robot collaborative mapping, Table 5 presents the time consumption comparison between single-robot mapping and collaborative mapping with two robots in the same scenario. In the identical experimental setting, constructing a global map solely relying on a single robot requires 69.596 s, whereas collaborative construction of the global map by two robots in the same scenario only takes 34.660 s. It is evident that the mapping efficiency improves by nearly 50% with two robots compared to a single robot. Experimental results conducted in real environments demonstrate that our algorithm achieves map fusion when two robots detect scene overlap. Compared to single-robot-mapping algorithms, our multi-robot collaborative mapping algorithm enhances mapping efficiency by nearly 50%, significantly reducing mapping time and enabling rapid mapping in large-scale environments.

## 6. Conclusions

Proposal of a Multi-Robot Collaborative Mapping Visual SLAM Algorithm Integrating Point-Line Features addressing the inadequate performance of single mobile robots in indoor environments with weak textures and artificial structured scenes relying solely on feature-based visual SLAM, this paper presents a multi-robot collaborative mapping visual SLAM algorithm that incorporates point-line features. Considering the research objectives and laboratory conditions, the ORB-SLAM3 algorithm, which is well-established in the current research, is chosen as the foundational framework. The frontend visual odometry feature extraction is enhanced by integrating line features into point features to enrich the features extracted in environments with distinct weak-textured structures. Experimental validation demonstrates the feasibility and robustness of the improved algorithm, which is extended to multi-robot scenarios. A multi-robot collaborative mapping algorithm based on scene recognition is proposed to enhance mapping efficiency for larger-scale indoor scenes. However, depth cameras exhibited reduced performance when mapping environments with frequent changes in object positions, such as in crowded or cluttered indoor spaces. Edge noise near walls resulted in artifacts in the generated point clouds, impacting the quality of the maps. IMUs were useful for capturing quick movements but introduced noticeable drift over longer periods, particularly when the robot moved through large, featureless indoor spaces. LiDAR can improve the efficiency of mobile robots in mapping edge areas, but LiDAR data showed inaccuracies when mapping areas with highly reflective surfaces, such as glass walls, where reflections led to erroneous distance readings. Therefore, when facing complex problems, considering the adoption of multi-sensor fusion algorithms, including depth camera, IMU, and LiDAR, for collaborative mapping can improve mapping accuracy.

## Figures and Tables

**Table 1:** Accuracy results of the algorithm on the Structure_texture dataset.

Alg	Max	Mean	Median	Min	RMSE	Sse	Std
ORB-SLAM3	0.104	0.066	0.065	0.025	0.069	4.296	0.021
ORB-SLAM2	0.049	0.019	0.018	0.003	0.020	0.350	0.006
Proposed	0.123	0.056	0.051	0.008	0.061	3.410	0.025

**Table 2:** Accuracy results of the algorithm on the Nostructure_notexture dataset.

Alg	Max	Mean	Median	Min	RMSE	Sse	Std
ORB-SLAM3	0.131	0.067	0.064	0.051	0.068	0.503	0.012
ORB-SLAM2	0.101	0.052	0.049	0.038	0.053	0.318	0.011
Proposed	0.075	0.034	0.031	0.023	0.035	0.113	0.009

**Table 3:** Accuracy results of the algorithm on the Structure_notexture dataset.

Alg	Max	Mean	Median	Min	RMSE	Sse	Std
ORB-SLAM3	0.097	0.081	0.081	0.066	0.081	1.665	0.006
ORB-SLAM2	0.101	0.083	0.083	0.065	0.084	1.777	0.006
Proposed	0.065	0.035	0.036	0.007	0.036	1.045	0.010

**Table 4:** Comparison of RMSE of algorithms.

Alg	Str_notext_far	Str_notext_near
ORB-SLAM3	0.081	0.0192
ORB-SLAM2	0.084	0.0212
Proposed	0.036	0.0158

**Table 5:** Robot build time comparison (s).

Number of Mapping Machines	Mapping Time
Individual robot	69.596
Two robots	34.660

## Data Availability

Data are contained within the article.
